# OA03.04. An assessment of the effectiveness of acupuncture for the Trauma Spectrum Response: results of a rapid evidence assessment of the literature (REAL)

**DOI:** 10.1186/1472-6882-12-S1-O12

**Published:** 2012-06-12

**Authors:** C Lee, J Smith, M Sprengel, C Crawford, D Wallerstedt, R Welton, A York, A Duncan, W Jonas

**Affiliations:** 1 Samueli Institute, Alexandria, USA; 2 Healingworks: Restoring and Renewing Military Families, Silver Spring, USA

## Purpose

Exposure to deployment, combat, and other traumatic events can induce a spectrum of physical, cognitive, psychological and behavioral effects. This constellation has been termed the “Trauma Spectrum Response” (TSR) and includes depression, anxiety, post-traumatic stress disorder (PTSD), traumatic brain injury (TBI), sleep disturbances, substance abuse, cognition, headache, pain, and spirituality. Acupuncture has been shown to effectively treat such symptoms as headache, insomnia, stress, anxiety, depression, and perceived pain. While many literature reviews on acupuncture have been published, their conclusions are often difficult to interpret, inconsistent or contradictory. Thus, in order to comprehensively assess acupuncture’s effectiveness across these symptoms, a rapid evidence assessment of the literature (REAL) was conducted to rigorously and systematically assess the quantity and quality of the existing systematic reviews/meta-analyses to gauge the effectiveness of acupuncture in treating the various components of TSR.

## Methods

We conducted a search of systematic reviews published in the English language in several key databases (PubMed, Cochrane Database of Systematic Reviews, EMBASE, PsycINFO, and CINAHL) from inception to September 2011. Articles were included if they involved needle acupuncture as a treatment for at least one of the TSR components.

## Results

We present the results of this review of systematic reviews for each of the TSR components and discuss the overall quality of these reports using the Scottish Intercollegiate Guidelines Network (SIGN) 50. Additionally, we present the results on the overall effectiveness of acupuncture and safety rating for each of the TSR components as determined by a group of subject matter experts using a systematic grading methodology.

## Conclusion

The results of this review of reviews is expected to provide a systematic assessment of the quality of the current body of acupuncture literature for each of the TSR components and identify gap areas that may elicit further research.

